# Investigation on prevalence and risk factors associated with genitourinary syndrome of menopause in middle-aged and older women in Beijing community: a cross sectional study

**DOI:** 10.1186/s12905-022-02099-w

**Published:** 2022-12-30

**Authors:** Ye Zhu, Junxiu Wei, Xin Yang, Wei Zhu, Weiting Zhang

**Affiliations:** 1 Department of Obstetrics and Gynecology, Peking University People’s Hospital, Xicheng District, No. 11, Xizhimen South Street, Beijing, 100044 China; 2 Department of Obstetrics and Gynecology, Affiliated Hospital of Hebei University, Baoding, China; 3 Mudanjiang Medical University, No.3 Tong Xiang Street, Mudanjiang City, 157011 China

**Keywords:** Genitourinary syndrome of menopause, Pelvic organ prolapse, Risk factor, Urinary incontinence, Vulvovaginal atrophy

## Abstract

**Background:**

Genitourinary syndrome of menopause (GSM) comprises genital symptoms (dryness, burning, itching, irritation, bleeding), sexual symptoms (dyspareunia and other sexual dysfunctions) and urinary symptoms (dysuria, frequency, urgency, recurrent urinary infections) associated with menopause. To avoid invasive testing and painful physical examinations, validated questionaries, which can assess the prevalence and risk factors associated with symptoms of GSM. We aimed to investigate the prevalence and risk factors associated with GSM in middle-aged and older women in the communities of Beijing, China.

**Methods:**

A cross-sectional, questionnaire study was performed among 35–70 years old Chinese woman. Vaginal health index score and urinary distress inventory (UDI-6) was used to evaluate vulvovaginal atrophy (VVA) and urinary incontinence (UI). Stages of pelvic organ prolapse (POP) was measured during gynecological examination with POP-Q system. Mean ± standard deviation (SD) and proportion/percentages were used to summarize continuous and categorial variables respectively**.** The Bonferroni method was used to adjust for multiple comparisons.

**Results:**

A total of 2702/3000 participants completed the questionnaire survey. The mean ± SD age of participants was 53.7 ± 7.0 years and prevalence of VVA among participants was 34.8% (941/2702). In UDI-6 questionnaires total 47.5% (1284/2702) participants reported experiencing urinary incontinence (UI). Further, POP was highly prevalent in anterior vaginal wall 38.9% (1050/2702) followed by posterior vaginal wall 25.3% (683/2702) and uterine 22.2% (599/2702). Besides, multiple logistic regression analysis inferred older age (45–54 years [OR (95% CI): 3.38 (2.03, 5.64)]; 55–64 years [OR (95% CI): 8.63 (5.09, 14.64)]), menopause [OR (95% CI): 2.20 (1.71, 2.85)] and Faecal Inconsistence **(**FI) [OR (95% CI): 1.31(1.00, 1.72)] as independent risk factors for VVA.

**Conclusions:**

Our study evidenced that GSM is prevalent in old age Chinese women. GSM is related with UI, POP and VVA. Further older age, menopause and FI were risk factors associated with VVA. Our findings could help health care personnel to get a comprehensive overview of factors associated with VVA and urinal distress, which may facilitate early detection and prevention of GSM.

## Background

Menopause is a condition resulting from estrogen deficiency [1]. The average age of natural menopause is 48.9 years in Chinese woman and it evidences that one-third of women's life is spent in menopause and post menopause [2]. General menopausal symptoms include physical, genitourinary and psychological symptoms, such as mood swings, hot flashes, bone and joint symptoms, and urogenital discomfort [3]. Genitourinary syndrome of menopause (GSM) is a relatively new term, first introduced in 2014 by the international society for the study of women’s sexual health and the North American menopause society and describes various menopausal symptoms [4]. The new definition of GSM comprises genital symptoms (dryness, burning, itching, irritation, bleeding), sexual symptoms (dyspareunia and other sexual dysfunctions) and urinary symptoms (dysuria, frequency, urgency, recurrent urinary infections) associated with menopause [5]. It describes the constellation of lower urogenital tract signs and symptoms associated with a low-estrogen state, such as vulvovaginal atrophy (VVA) and urinary incontinence (UI). GSM have great impact on both premenopausal & postmenopausal women health but it mostly occurs during the menopausal transition [6, 7].

The prevalence of symptoms of GSM has been reported in about 30% of Chinese mid-life women [8]. Further, diagnosis of GSM is challenging because, 50% of postmenopausal women with mild or moderate GSM are asymptomatic [9]. It has been documented that UI and VVA are directly associated with GSM [10, 11]. Moreover, GSM increases the likelihood of pelvic organ prolapse (POP) (ureteral and vaginal prolapse) [12]. GSM is still an under-reported problem because of limitations of previous studies like limited sample size, specific ethnical homogeneity of participants, biased selection criteria (general women were not examined only gynaecological OPD patients were examined), and hard to recruit women having an interest in study participation [7, 13]. Besides, diagnosis of GSM includes physical pelvic examination, vaginal pH and invasive testing such as vulval, vaginal and urethral anatomical biopsies [14]. To avoid invasive testing and painful physical examinations, validated questionaries, which can assess the prevalence and risk factors associated with symptoms of GSM are much warranted [7]. Early detection of GSM can avoid the long-term risks and complications that may severely compromise quality of life and sexuality of a women [15]. There are few large scale sample epidemiological studies on GSM in Chinese population [14, 16]. Therefore, the current study was aimed to investigate the prevalence and risk factors associated with GSM in middle-aged and older women in the communities of Beijing, China.

## Methods

### Study design and participants

In this cross-sectional study, Chinese woman (35–70 years of age) who voluntarily participated in cervical cancer and breast cancer screening tests within the administrative division of Beijing No. 2 Hospital and the Yuetan Community Health Service Center under the Fuxing Hospital of Capital Medical University were invited for a questionnaire survey from October to December 2016. After informing the content and significance of the study, a written informed consent according to the Declaration of Helsinki was obtained from the participants. The study was approved by the medical ethics committee of the Peking University People's Hospital (Code number: 2015PHB087-01).

### Study measures

#### Socio-demographic survey questionnaire

The study questionnaires collected the information about socio-demographic characteristics of participants including age, educational level, menstrual status/condition, menopausal hormone replacement therapy (HRT), physical disease (high blood pressure, heart disease, hyperthyroidism, hypothyroidism, Diabetes Mellitus, arthritis, dyslipidemia, tumor), gynaecological surgery, body mass index (BMI), parturition mode and income level by certified medical staff.

#### Assessment of GSM-questionnaire

A validated and simple questionnaire was used to asses GSM. The following associated symptoms were assessed.

*Menopause/perimenopause* We defined ‘menopause’ as 12 months of amenorrhea (without any other explanations). Perimenopause is considered as persistent difference of 7 or more days in the length of consecutive cycles (persistence meant 10-cycle- recurrence from the first variable length cycle) [17, 18].

*Vaginal health index* Vaginal health index score (VHIS) was evaluated based on following parameters: elasticity, fluid volume, pH, epithelial integrity and moisture. The score of each parameter varied between 1 (worst condition) and 5 (best condition), the maximum possible score was 25 and the minimum possible score was 0. If the total score is < 15, it is considered that there is vulvovaginal atrophy (VVA) [19]. Participants were also inquired about the presence of VVA symptoms: vaginal pain, dyspareunia, vaginal pruritus and vaginal burning [1].

*Urinary distress continence* Urinary distress was evaluated by UDI-6 [20, 21] UDI-6 consists of 6 questions: 1 – Frequent urination, 2 – Leakage related to feeling of urgency, 3 – Leakage related to activity, 4 –Coughing, or sneezing small amounts of leakage (drops), 5 – Difficulty emptying the bladder, and 6 – Pain or discomfort in the lower abdominal or genital area. Based on above questions, Urinary incontinence was classified into three categories: Stress urinary incontinence (SUI) - (questions 3 and 4), Urgency urinary incontinence (UUI) (questions 1 and 2) and Mixed Urinary Inconsistence (MUI)- (questions 1,3 and 5,6).

*Faecal Inconsistence (FI)* Fecal incontinence was assessed by following questions: (1) Do you usually lose stool beyond your control if your stool is well formed? (2) Do you usually lose stool beyond your control if your stool is loose? (3) Do you usually lose gas from the rectum beyond your control? Subjects responded with either “none (0), slight (1), moderate (2), or greatly (3)” [22].

*Pelvic organ prolapses (POP)* POP refers to descent of one or more of anterior vaginal wall, posterior vaginal wall, the uterus (cervix), or the apex of the vagina. The POP stage of the participants was evaluated during gynecological examinations via lithotomy position by Pelvic Organ Prolapse Quantification system (POP-Q [23, 24]. We defined POP as Stage I–IV, which meant the most distal portion of the prolapse was > 1 cm above the level of the hymen (i.e., its quantitation value was < − 1 cm). The investigators were trained to form a professional team familiar with the contents of questionnaire and POP-Q system.

The questionnaires took approximately 20 min to complete, and the investigators checked the questionnaires after collection to guarantee the quality of the responses.

## Statistical analysis

Mean ± standard deviation (SD) and proportion/percentages were used to summarize continuous and categorial variables respectively. Comparison of two or more groups of categorial variables was performed by chi-square. *p* < 0.05 was considered as statistically significant. The Bonferroni method was used to adjust for multiple comparisons. When two of the three groups were compared, the Bonferroni method was used, and *p* < 0.001 was considered statistically significant. Multiple logistic regression analysis was used to analyze the risk factors of urinary distress and POP. SPSS 19.0 software was used for statistical analysis of data.

## Results

A total of 3000 women participated in this survey out of which 2702 completed the questionnaire (response rate 90.1%), 107(3.6%) copies of questionnaire were missing due to human or system errors, 11(0.4%) copies were excluded because participants were not ready to disclose the GSM results, 91 (3.0%) participants were rejected because they had undergone hysterectomy and 89 (2.9%) participants were excluded because they had undergone both modes of parturition (vaginal and caesarean section).

### Demographic characteristics

The demographic characteristics of the participants are presented in Table 1. The mean age of participants was 53.7 ± 7.0 years. More than half of the participants were menopausal (58.5%) and the mean age of menopausal women was 50.0 ± 3.1 years. All the participants had not undergone any surgery or treatment for POP.

**Table 1:** Basic demographic characteristics of participants

Baseline characteristic	N (%)
Age (years) (N)	2644
35–44 years	329 (12.4)
45–54 years	972 (36.8)
55–64	1329 (50.3)
≥ 65	14 (0.5)
Physical disease* (N)	2702
Yes	1551 (57.4)
No	1151 (42.6)
Gynecological disease (N)	2702
Yes	2088 (77.3)
No	614 (22.7)
HRT (menopause drugs) (N)	2702
Yes	598 (22.1)
No	2104 (77.9)
Gynecological surgery (N)	2702
Yes	372 (13.8)
No	2330 (86.2)
Menstrual status (N)	2638
Regular	835 (31.7)
Irregular	261 (9.9)
Menopause	1542 (58.5)
BMI (kg/m^2^) **	2636
< 18.5	176 (6.7)
18.5–24.9	1897 (72.0)
≥ 25	563 (21.4)
Education level (N)	2644
Junior high school and below	1118 (42.3)
High school and secondary school	910 (34.4)
College or bachelor degree	616 (23.3)
Mode of parturition (N)	2702
Vaginal	1902 (70.4)
Caesarean	686 (25.4)
Nulliparous	114 (4.2)
Number of pregnancies(N)	
0	101 (3.7)
1	1421 (52.6)
2	675 (25.0)
≥ 3	505 (18.7)

*BMI*, Body mass index; *HRT*, Hormone replacement therapy

*Physical disease included: high blood pressure, heart disease, hyperthyroidism, hypothyroidism, Diabetes Mellitus, arthritis, dyslipidemia, tumor and so on. **BMI: Body Mass Index

### Prevalence and risk factors associated with symptoms of genitourinary GSM

The mean VHIS score of the participants was 15.1 ± 3.4 (elasticity 2.97 ± 0.77, moisture 2.98 ± 0.76, pH 3.02 ± 0.73, epithelial integrity 3.11 ± 0.68, fluid volume 3.00 ± 0.79). Overall, 34.8% (941/2,702) of the participants enrolled in the study suffered from VVA and the most prevalent symptoms of VVA includes vaginal pain 8.2% (222/2702), dyspareunia 15.5% (418/2702), vaginal pruritus 19.7% (533/2702) and vaginal local burning sensation 8.0% (215/2702). Further comparison of the VVA symptoms based on menopausal stage (Table 2) shown that prevalence of dyspareunia (17.8%) as most significant VVA symptom in menopausal women when compared to pre-menopausal (11.0%) or peri menopausal women (16.1%), *p* < 0.001. Moreover, multiple logistic regression analysis (Table 3) inferred older age (45–54 years [OR (95% CI): 3.38 (2.03, 5.64)], *p* < 0.001; 55–64 years [OR (95% CI): 8.63 (5.09, 14.64)], *p* < 0.001); menopause [OR (95% CI): 2.20 (1.71, 2.85)], *p* < 0.001; gynecological diseases [OR (95% CI): 1.61(1.28, 2.03)], p < 0.001 and higher education level (College or bachelor degree[OR (95% CI): 1.70 (1.30, 2.23)], *p* < 0.001) as significant risk factors for VVA.

**Table 2:** Frequency of symptoms of VVA among different stages of menopause

Symptoms of VVA	Pre menopause N = 835 (%)	Perimenopause N = 261 (%)	Menopause N = 1542 (%)	X^2^	p*
Vaginal pain	61 (7.3)	19 (7.3)	139 (9.0)	2.475	0.290
Dyspareunia	92 (11.0)	42 (16.1)	275 (17.8)	19.286	< 0.001
Vaginal pruritus	162 (19.4)	63 (24.1)	304 (19.7)	3.048	0.218
Vaginal burning	56 (6.7)	25 (9.6)	133 (8.6)	3.510	0.173

*VVA*, Vulvovaginal atrophy

* p < 0.05 was considered as statistically significant. The Bonferroni method was used to adjust for multiple comparisons and *p* < 0.001 was considered statistically significant

**Table 3:** Multivariate logistic regression analyses of risk factors for VVA

Exposure variables	B	SE	Adjusted OR*	95% CI	*p*
*Age(years)*					
35–44			Reference		
45–54	1.288	0.251	3.386	2.032, 5.641	< 0.001
55–64	2.303	0.258	8.635	5.090, 14.649	< 0.001
≥ 65	1.719	0.625	3.636	0.965, 13.707	0.057
*Menstrual status*					
Regular			Reference		
Irregular	0.124	0.19	1.055	0.711, 1.564	0.792
Menopause	0.748	0.123	2.209	1.710, 2.852	< 0.001
*Gynecological surgery*					
No			Reference		
Yes	-0.502	0.135	0.642	0.489, 0.843	0.001
*Gynecological diseases*					
No			Reference		
Yes	0.612	0.113	1.618	1.289, 2.030	< 0.001
*Education level*					
Junior high school and below			Reference		
High and secondary school education	0.437	0.103	1.501	1.216, 1.853	< 0.001
College or bachelor degree	0.479	0.131	1.709	1.305, 2.238	< 0.001
Fecal incontinence (FI)**					
No			Reference		
Yes	0.016	0.094	1.312	1.001, 1.720	0.049

*B*, Beta values; *SE*, Standard of error; *OR*, odds ratio; *CI*, confidence interval

*The OR was adjusted for all variables

Furthermore, among the 2702 subjects, 47.5% (1284/2702) experienced UI, among whom 24.8% (671/2702) experienced UUI, 40.1% (1084/2702) experienced SUI and 18.8% (508/2702) reported MUI. Among the urinary distress symptoms, the top three were urine leakage related to coughing, sneezing, or laughing (40.1%; 1084/2702), frequent urination (27.4%; 741/2702), urine leakage associated with a feeling of urgency (24.8%; 671/2702). In addition, 12.3% (333/2702) reported experiencing FI. Besides, multiple logistic regression analysis (Table 4) demonstrated that physical disease [OR (95% CI) 1.538 (1.295, 1.8280], *p* < 0.001; menopause drugs application [OR (95% CI): 1.79 (1.44, 2.22)], *p* < 0.001 and FI [OR (95% CI): 8.303(5.55, 12.40)], *p* < 0.001 were significant risk factors for urinary distress.

**Table 4:** Multivariate logistic regression analyses of risk factors for urinary distress

Exposure variables	B	SE	Adjusted OR*	95% CI	*p*
*Mode of parturition*					
Vaginal delivery			Reference		
Caesarean section	− 0.307	0.101	0.723	0.596, 0.879	0.001
Nullipara	− 0.656	0.231	0.67	0.412, 1.090	0.107
*Physical disease*					
No			Reference		
Yes	0.423	0.089	1.538	1.295, 1.828	< 0.001
*HRT (menopause drugs)*					
No			Reference		
Yes	0.604	0.11	1.792	1.441, 2.228	< 0.001
*Gynecological diseases*					
No			Reference		
Yes	0.143	0.105	1.259	1.033, 1.535	0.023
*Education level*					
Junior high school and below			Reference		
High school and secondary school education	− 0.182	0.101	0.737	0.606, 0.897	0.002
College or bachelor degree	− 0.143	0.115	0.833	0.667, 1.039	0.105
*Pelvic organ prolapse*					
No			Reference		
Yes	1.018	0.14	1.195	1.007, 1.419	0.042
*Fecal incontinence*					
No			Reference		
Yes	1.516	0.101	8.303	5.555, 12.409	< 0.001

*B*, Beta values; *SE*, Standard of error; *OR*, odds ratio; *CI*, confidence interval

*The OR was adjusted for all variables

Additionally, we investigated the prevalence of different types of POP among participants and the results obtained shown higher prevalence of anterior vaginal wall prolapse 38.9% (1050/2702) followed by posterior vaginal wall prolapse 25.3% (683/2702) and uterine prolapse 22.2% (599/2702). Further, comparison based on parturition modes (Table 5) shown that the prevalence of POP is higher in cesarean section group 47.7% (327/686) as compared to vaginal delivery group or nullipara group.

**Table 5:** Comparison of Pelvic Organ Prolapse in different parturition modes

Types of POP	Vaginal delivery N = 1902	Caesarean section N = 686	Nulliparous N = 114	X2	p*
Uterine prolapse	428 (22.5)	157 (22.9)	14 (12.3)	6.788	0.034
Anterior vaginal wall prolapses N (%)	760 (40.0)	270 (39.4)	20 (17.5)	22.839	< 0.001
Posterior vaginal wall prolapses N (%)	485 (25.5)	179 (26.1)	19 (16.7)	4.766	0.092
Total	884 (46.5)	327 (47.7)	27 (23.7)	23.776	< 0.001

*POP*, Pelvic organ prolapse

*p < 0.05 was considered as statistically significant. The Bonferroni method was used to adjust for multiple comparisons and *p* < 0.001 was considered statistically significant

## Discussion

To the best of our knowledge, this is the largest cross- sectional study to evaluate the prevalence and risk factors associated with symptoms of GSM in Chinese women of Beijing community. Current study evidenced that 34.8% of the enrolled participants suffered from VVA. Further, comparison of the VVA symptoms based on menopausal status revealed dyspareunia (17.8%) as most significant VVA symptom in menopausal women when compared to pre-menopausal (11.0%) or peri menopausal women (16.1%) *p* < 0.001. With an exception of study population from different regions these findings were in agreement with earlier studies reporting the prevalence of VAA. Earlier, Nappi et al., conducted a structured online questionnaire study by including around 3520 postmenopausal women aged 55–65 years and reported 45% prevalence of VAA [25]. Later, Lin et.al conducted a retrospective study involving 3450 perimenopausal women reported a prevalence of VVA at 12.4% [26]. The prevalence of VVA in the current survey was higher than the Lin’s study, which might be attributed to the participants “older age’. Besides, this finding could be attributed to those women unable to discuss about VAA because of personal embarrassment and cultural reasons. Further, the expression of "GSM" is more implicit than the word "VVA", which is more conducive to patients seeking for treatment. Further, in the current study we have found postmenopausal women were more likely to develop VVA, which may be due to the different composition of the vaginal microbiota in the vagina and estrogenic effect on vaginal environment.

Also, UDI-6 can accurately and credibly assess the severity of urinary distress and their impacts on the quality of life of pelvic floor disorder women, as well as assess the general women [27, 28]. In the current study, around 47.5% experienced UI and the results obtained were lower than the previous reported studies where, Gyhagen et al. conducted a registry-based national cohort study by including 5236 participants reported that 63% women had UI [29]. These differences might be due to sample size or due to inclusion of study population from different regions. Moreover, in the present study among all symptoms, SUI (40.1%), occurred more frequently. However, earlier studies reported the SUI occurs at a frequency of 22–25% [30, 31]. The reason for this disparity is that in our study, we were aiming at middle-aged and older women, where as other studies has included women aged over 20 years. In addition, aging and age-related changes in the bladder might play a significant role in the development of UI.

Similarly, delivery mode had a significant impact on the UI, which might be attributed to more damage of pelvic floor tissue caused by vaginal delivery. Our study evidenced that cesarean section as protective factor for urinary distress [7]. These findings are in agreement with earlier reports where, Rortveit’s et al. [32] reported that the risk of UI was higher among women who had vaginal deliveries than among women who have had cesarean sections. Moreover, multivariate logistic regression analysis revealed that physical disease, gynecological diseases, menopause drugs application, POP and FI as independent risk factors for urinary distress. These findings are in consistent with previous reports where, Legendre et.al conducted a review of literature dealing with the epidemiology of UI in women and reported that oral HRT had a detrimental effect on SUI and had less effect on UI [33]. In addition, Zhu et al. [34], conducted a cross-sectional survey with approximately, 20,000 Chinese women reported that perimenopause and post menopause status were potential risk factors for SUI. Moreover, recent studies reported that the presence urinary incontinence increases the risk for fecal incontinence ranging between 2-and 4-fold [35].

Despite these noteworthy observations, the current study has certain limitations. Firstly, the study lacks temporality due to cross-sectional design which limits causal inference. Therefore, longitudinal studies are needed for clarification. Secondly, we selected only two communities in Beijing, hence limited sample size could not completely represent the situation of all women in Beijing and limits the generalizability of results.

To conclude the current study provided an insight on the factors associated with GSM. Similar studies are needed to further investigate the factors associated with prevalence of GSM in women from all provinces of China.

## Conclusion

VVA was found to be more prevalent (34.8%) among middle-aged and older Chinese women. POP was highly prevalent in interior vaginal wall followed by posterior vaginal wall. Older age, menopause, higher education level and gynecological diseases were found to be significant risk factors and dyspareunia was most significant symptom associated with VVA. Our findings would help health care personnel to communicate easily with patients and improve their understanding about GSM. Clinicians can enquire about the GSM risk factors during routine gynecological examination, which would help in early diagnosis and treatment of GSM.

## Data Availability

The datasets generated during and/or analyzed during the current study are available from the corresponding author on reasonable request.
